# Quantifying impacts of white-tailed deer (*Odocoileus virginianus* Zimmerman) browse using forest inventory and socio-environmental datasets

**DOI:** 10.1371/journal.pone.0201334

**Published:** 2018-08-23

**Authors:** Stephanie R. Patton, Matthew B. Russell, Marcella A. Windmuller-Campione, Lee E. Frelich

**Affiliations:** Department of Forest Resources, University of Minnesota, St. Paul, Minnesota, United States of America; Universita degli Studi di Sassari, ITALY

## Abstract

Elevated population levels of white-tailed deer (*Odocoileus virginianus* Zimmerman) can drastically alter forest ecosystems and negatively impact society through human interactions such as deer vehicle collisions. It is currently difficult to estimate deer populations at multiple scales ranging from stand, county, state, and regional levels. This presents a challenge as natural resource managers develop silvicultural prescriptions and forest management practices aimed at successfully regenerating tree species in the face of deer browsing. This study utilized measurements of deer browse impact from the new tree regeneration indicator developed by the United States Department of Agriculture Forest Service Forest Inventory and Analysis (FIA) program. Seedling and sapling abundance and other plot-level characteristics were analyzed across three states (Michigan, Minnesota, and Wisconsin) in the Great Lakes Region of the United States. Socio-environmental datasets (Lyme disease cases, deer vehicle collisions, and deer density estimates) were used in conjunction with FIA data to determine their predictive power in estimating deer browse impacts by county. Predictions from random forests models indicate that using Lyme disease case reports, the number of deer-vehicle collisions, deer density estimates, and forest inventory information correctly predicted deer browse impact 70–90% of the time. Deer-vehicle collisions per county ranked highly important in the random forests for predicting deer browse impacts in all three states. Lyme disease cases ranked high in importance for the Lake States combined and for Minnesota and Wisconsin, separately. Results show the effectiveness of predicting deer browse impacts using a suite of freely available forest inventory and other socio-environmental information.

## Introduction

Selective browsing by white-tailed deer (*Odocoileus virginianus* Zimmerman) can have dramatic direct and indirect effects on forest ecosystems, including changes in the food web, vegetation structure, and nutrient cycling [1,2]. White-tailed deer consume buds and twigs of young trees as well as many understory herbaceous plants. Once the terminal leader of a tree grows above the reach of deer, impacts from browsing are significantly reduced–thus, seedlings and saplings are the most vulnerable to damage [3–5]. Ungulate browsing of select species for numerous years can greatly change forest species composition and diversity [2,6], depending on abundance of browse tolerant species (e.g. increased diversity [7,8]; decreased diversity [4,9,10]). In more extreme cases, deer alter forested ecosystems such that they exist in a different stable state. These studies depict a landscape typically more similar to grasslands and inhospitable for seedling establishment [11,12]. Understanding deer browse impact on forest structure and composition is critical to predicting the resiliency and productivity of forests in the long term [13].

Deer present additional challenges to forested communities across the eastern US via indirect effects. In a Pennsylvania exclosure study, deer promoted the success of two invasive species–garlic mustard (*Alliaria petiolata* ((M. Bieb.) Cavara & Grande)) and Asian stiltgrass (*Microstegium vimineum* (Trin.) A. Camus)–and reduced the success of native plants via selective browsing [14]. The community-wide effects can also be seen further up the trophic cascade. Shrub nesting birds decline in richness and abundance when deer densities exceed eight per square kilometer in Pennsylvania [15]. In oak forests, competition for acorn mast caused a reduction in small mammal populations [16,17]. High deer browse effects also extend beyond the forest ecosystem. Over time, loss of palatable tree species and proliferation of nonpalatable species potentially affects timber markets when merchantable species are affected [3,18].

Estimating deer densities and browse impacts are challenging at broad scales. One of the ways in which browse pressure might be predicted is with socio-environmental datasets that provide information related to deer-human encounters. White-tailed deer are the primary hosts for adult black-legged ticks (*Ixodes scapularis* Say), which can transmit Lyme disease (caused by the bacterium *Borrelia burgdorferi*) to humans [19]. Although other animals, such as the white-footed mouse (*Peromyscus leucopus*) and ground-dwelling birds host *I*. *scapularis* during its life cycle, adult female ticks detach from deer in the fall of their second year and typically lay several hundred to thousands of eggs, therefore deer ultimately determine the location of spring nymphs [20]. Although the relationship between deer and Lyme disease is complex, white-tailed deer populations and *I*. *scapularis* populations were correlated in a number of studies in the northeastern US [21–25]. Others found a decline in human Lyme disease infections with reduced deer populations [26] or correlations between tick populations and human cases of Lyme disease [27,28], which suggests that disease rates may help to estimate deer abundance. Deer also contribute to thousands of vehicle collisions per year, resulting in property damage, injury, and occasionally death [29]. In this analysis, we aim to use these datasets as surrogates for deer densities.

Deer populations in the Great Lakes Region of the United States (Lake States henceforth) have increased in recent decades, making them “hyperabundant” in many counties and a threat to regenerating tree species [30,31]. This increase in population is mostly due to habitat modification and reduction in predatory species in historical home ranges [30,32]. Regional deer populations are difficult and expensive to precisely estimate, especially across ownership and geographic boundaries. Feeding behaviors of deer cluster based on habitat quality, predator populations, and physical feature boundaries, supporting the use of a herbivory metric as opposed to deer density alone to determine effects of browsing across the landscape [13,30,33,34]. Prior to the regeneration indicator from the United States Department of Agriculture (USDA) Forest Service Forest Inventory and Analysis (FIA) program implementation in 2012, there has not been a nationally consistent classification of animal browsing [35]. Not only does this deer herbivory metric better capture the heterogeneity of deer browsing, the FIA program provides opportunities for analysis at the regional scale. Traditional large-scale studies of deer herbivory are limited by time and space or utilize deer densities as a proxy for deer browsing.

Predicting the complex nature of deer browse impacts may require a suite of information including deer density, tree seedling and sapling abundance, and socio-environmental variables. The objective of this study is to use publicly available socio-environmental datasets in combination with forest inventory data to predict deer browse pressure in forests across the Lake States. These publicly available datasets have not been utilized in this way before and would provide a simple alternative to complex deer models.

## Methods

### Study area

This study was conducted across the US Lake States–in the states of Michigan (MI), Minnesota (MN), and Wisconsin (WI). The Lake States contain a mixture of tallgrass prairie, temperate deciduous forests, and mixed boreal forest biomes that are defined by the Laurentian mixed forest, eastern broadleaf forest, and prairie parkland ecoregions. Wind and fire have historically influenced the forest composition and significant land clearing by European settlers began around 1880. Typical summers are short and cool with long and cold winters. Areas within 10-km of the Great Lakes experience a strong lake effect exhibiting higher temperatures in the winter and lower temperatures in the summer. Precipitation occurs mostly in the growing season with 800 to 900-mm annually. Forests of the Lake States contain diverse forest types and are comparable to many of the world’s forests found in cool-to-cold temperate zones [36,37].

### US Forest Inventory and Analysis data

The US Department of Agriculture Forest Service’s Forest Inventory and Analysis (FIA) program was the primary source of forest inventory data for this project. The FIA protocols are nationally consistent and provide a basis for comparison across regions of the United States [38]. This program divides the US into populations—(typically counties) and subpopulations. Each population and subpopulation has a defined number of plots based on forested area. There are three phases of plots in the FIA program, of which the first two are used in this analysis: Phase 1 (P1) and 2 (P2). Phase 1 stratifies the land into forest- (defined as an area of at least 0.4-ha in size, 36.6-m wide and 10% stocked with trees) or nonforest using aerial or satellite imagery. Phase 2 uses standard forest inventory methods to quantify structure, composition, and stand level attributes; plot density is approximately one plot for every 2,428-ha of forested land. A full description of the FIA stratification can be found in the USDA Forest Service national sampling design document [38].

Each P2 plot contains four subplots arranged as a central point with three points clustered around the center. The outer points lie 36.58-m from the center at an azimuth of 0, 120, and 240 degrees. Each point denotes the center of a fixed radius plot with a 7.32-m radius in which all trees 12.7-cm and larger in diameter at breast height (DBH) were measured. A microplot, located 3.66-m off center and at an azimuth of 90 degrees, has a 2.07-m radius in which saplings (2.54 to 12.7-cm DBH) and seedlings were measured. Within each microplot all live tree seedlings were tallied, where conifer and hardwood seedlings were at least 15.2 and 30.5-cm in height, respectively, with both having a DBH ≤ 2.54-cm.

On a subset of P2 plots, collection for regeneration indicator plots–named P2-plus plots–began in 2012 in the northern US. One plot was sampled every 19,425-ha of forest. In each plot, browse impact, which was quantified by visualizing direct browse–defined as consumption of shoots, twigs, and leaves by animals for food–and overall seedling abundance, was recorded on a scale of 1 through 5 (very low impact, low impact, medium impact, high impact, and very high impact, respectively) on seedlings at least 5.08-cm tall [35]. In total, 792 P2-plus plots were available from 2012–2015 (four years of data available from a five (MN) or seven (MI, WI) year cycle) in the Lake States. Field crews were thoroughly trained and plots were verified for quality control.

All FIA data were accessed via the “FIA DataMart” (www.fia.fs.fed.us/tools-data, downloaded 20 June 2016). Data are publicly and freely available. For the P2 plots, the PLOT, TREE, and SEEDLING tables were used to obtain information on live trees. For the P2-plus plots, deer browse impacts were obtained from the PLOT_REGEN tables (see S1 Table for county-level summary).

### Socio-environmental datasets

**Fig 1:**
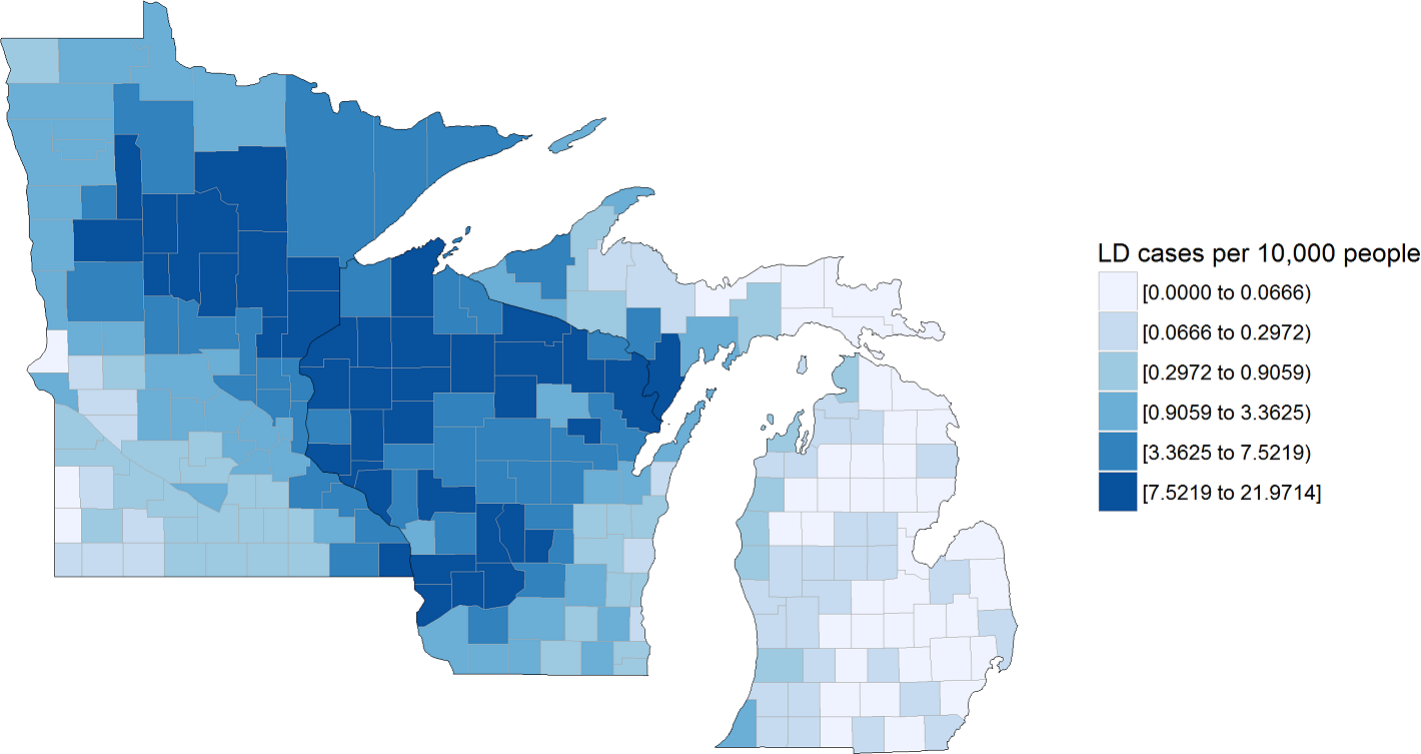
Average annual reported cases of Lyme disease (LD) for 2012–2015 in the US Lake States. Cases reported per 10,000 county residents.

**Fig 2:**
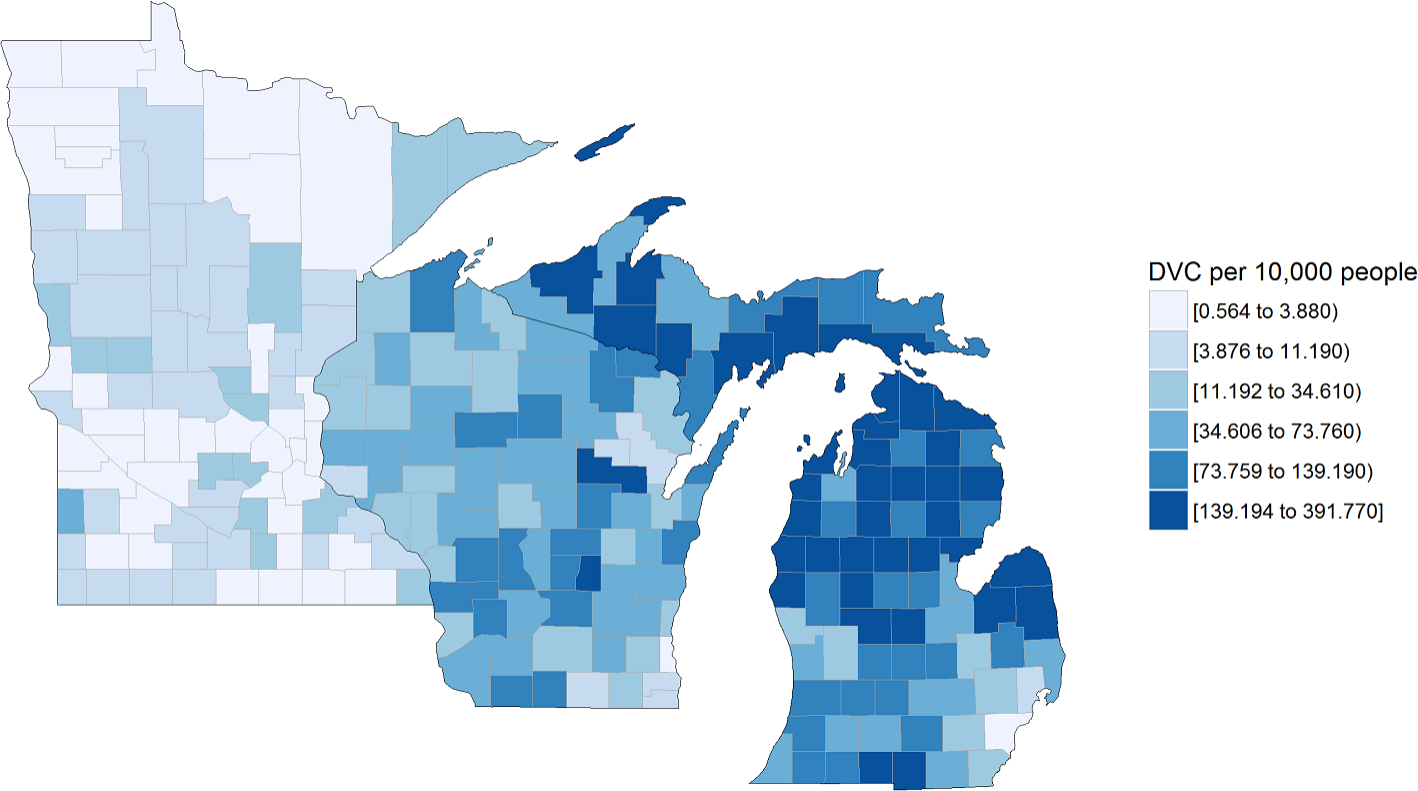
Average annual reported cases of deer-vehicle collisions (DVC) for 2012–2015 in the US Lake States. Cases reported per 10,000 county residents.

**Fig 3:**
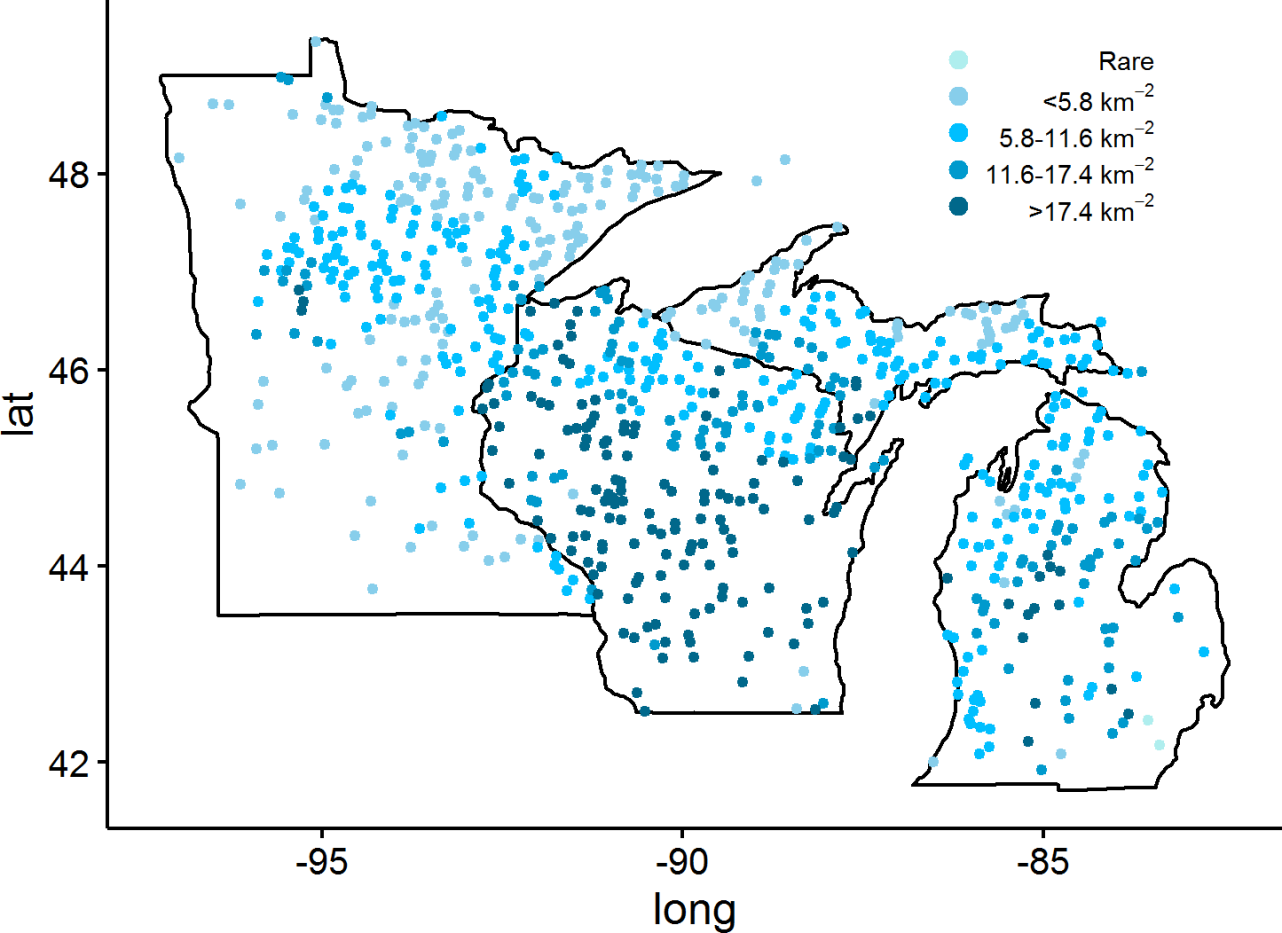
Location of P2-plus plots (n = 786, 2012–2015) within the US Lake States with corresponding deer density. Deer density provided by QDMA. Plots located outside the delineated state borders occurred on islands of the Lake States.

Socio-environmental datasets were used to determine their predictive power in estimating deer browse effects in forest ecosystems. These datasets included the Centers for Disease Control and Prevention (CDC) Lyme disease surveillance data (Fig 1), deer-motor vehicle crashes (Fig 2), and Quality Deer Management Association (QDMA) deer density estimates (Fig 3) [39,40]. Census data were used in the analysis of these datasets (human populations per county) to standardize these population-dependent variables per 10,000 residents (http://www.census.gov/popest/data/index.html).

Reported CDC Lyme disease cases have been described since 1991 and are based on county of residence. A case is counted if a physician identifies a patient with *erythema migrans*, which is a type of skin lesion that occurs in 60–80% of patients with Lyme disease (http://www.cdc.gov/lyme/stats/). A physician can conclude a case as suspected, plausible, or confirmed based on symptoms and exposure. Reported Lyme disease cases from 2012 to 2015 were used in this analysis.

Deer-vehicle collision reports were obtained from the Michigan State Police (http://www.mlive.com/news/index.ssf/2013/10/database_see_where_in_michigan.html), Minnesota Department of Public Safety (https://dps.mn.gov/divisions/ots/reports-statistics/Pages/Fact-sheets.aspx), and Wisconsin Department of Transportation (http://wisconsindot.gov/Pages/safety/education/crash-data/crashfacts.aspx). Total annual deer-vehicle collisions by county were obtained from these sources for 2012 through 2015.

Deer density estimates were obtained from the QDMA spatial map [40]. This map provides deer density estimates based on state agency reported information from 2001–2005. Plot level tree response to browsing represents historical browsing intensity, therefore the deer density estimates from approximately 10 years earlier are appropriate for use with recent FIA data [5]. There were five categories for deer density, based on deer per square kilometer: rare, less than 5.8 deer km^-2^, 5.8 to 11.6 deer km^-2^, 11.6 to 17.4 deer km^-2^, more than 17.4 deer km^-2^ [39].

### Models of deer browsing impacts

The R computational software was utilized in creating statistical models [41]. All analyses and comparisons occurred at the county or plot level. Tree stand and stocking information was summarized using FIA data to provide trees per hectare (TPH) for seedlings, saplings, and overstory trees and overstory basal area per hectare (BA m^2^ha^-1^) at the plot level. Overstory TPH and basal area provide a metric of current stand conditions which are important to understand when considering stand dynamics, especially regeneration. These variables could help explain discrepancies in seedling and sapling abundance, which are expected to be negatively affected by deer browsing, if palatable [2]. The combination of these explanatory variables was used in the analysis, because they capture forest density and natural regeneration appropriately.

All models were created using the randomForest package [42,43], which was implemented in R and utilizes classification trees of bootstrapped samples to determine variable importance [44]. Variable importance–defined as the mean decrease in error of a particular variable when included in the model–was used to determine the most important variables in models predicting deer browsing for forests across the region based on Lyme disease cases, deer-vehicle collisions, deer density data, and forest inventory variables (ie. BA, TPH). Phase 2-plus plots from 2012–2015 with forest inventory data, all socio-environmental datasets, and plots that were not located inside an exclosure were used in the analysis (n = 786). With this package, bootstrapped estimates of classification trees were estimated and 20% of the observations from the dataset were used to compare model performance.

Additionally, reduced models were created by eliminating the least important variables and running the random forests model again until the three most important variables were identified. To validate model accuracy, 20% of the plots were randomly sampled and predicted browse score was compared to the field-assessed browse score. This process was replicated 25 times for each model to determine model accuracy and an estimate of error.

Forest inventory variables utilized in the random forests analysis included seedling TPH, sapling TPH, overstory TPH, and overstory BA. Socio-environmental variables included collisions per county per 10,000 people, Lyme disease cases per county per 10,000 people, and QDMA deer density estimates.

The full model, utilizing all explanatory variables, was used to predict browse scores for all FIA plots in the Lake States (P2, n = 12,386) that were inventoried from 2012–2015. Predicted browse scores for all FIA plots were analyzed in ArcMap to create a map of browse score using inverse distance weighting (IDW) [45]. The IDW tool in ArcMap weights measured values that are nearest to each other to predict values at unmeasured locations. This interpolation accounts for the distance between points as well as spatial arrangement of the measured points. The result is a graphical representation of deer browse severity across the study region. We present the results as quantiles, as the interpolation will use an ordinal variable to interpolate continuously between categories and therefore defining appropriate breaks in browse impact score to correspond with the original categories is not possible.

## Results

### Socio-environmental and deer browsing trends

**Fig 4:**
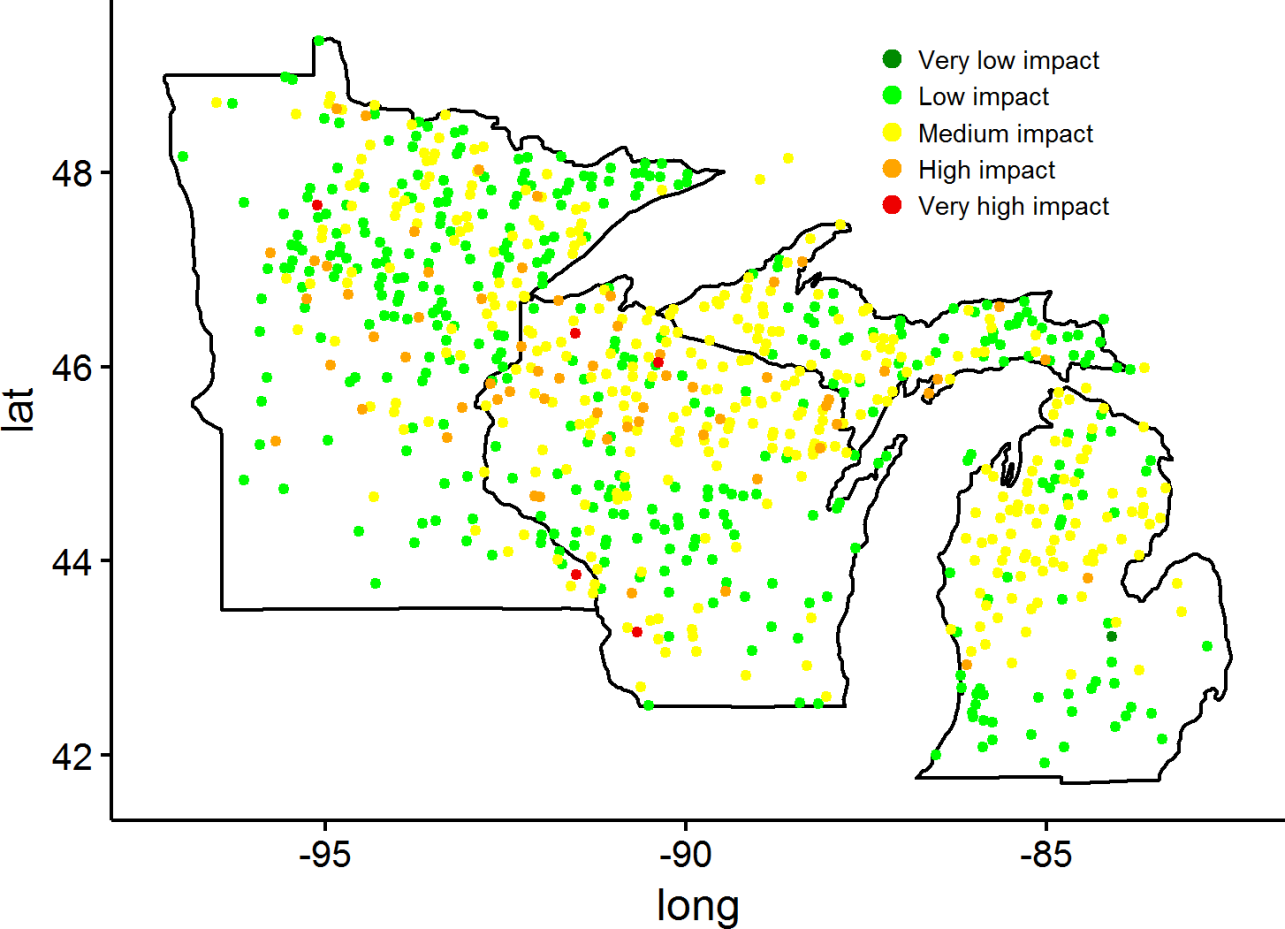
Location of P2-plus plots (n = 792) within the Lake States with browse impact score, 2012–2015. Plots located outside the delineated state borders occurred on islands of the Lake States.

Forest inventory plots with browse scores (n = 786) were distributed across the forested portions of the Lake States, with a lower plot density in non-forested regions such as in northwest and southwest Minnesota, and southeast portions of Wisconsin and Michigan (Fig 4). Some level of browsing occurred in more than half of the plots in Michigan and Wisconsin–medium impact on 54.6% and 47.9% of plots and high or greater on 3.4% and 14.0% of plots, respectively. In Wisconsin, most of the high and very high browse impacted plots occurred in the northern half of the state. Michigan had some areas with high deer browse impact in the Upper Peninsula as well as in the northern half of the Lower Peninsula. Compared to Michigan and Wisconsin, Minnesota had a higher proportion of plots with low browse impact (60.8% of plots) and the most plots of all three of the states (n = 288). Minnesota displayed diverse browse impacts across the landscape, with most of the medium or greater browse impacted plots located in central and northern Minnesota. Few FIA plots (n = 5) were classified as having very high browse impact. These plots were located in Minnesota and Wisconsin, one in the north-central part of Minnesota, two in the north, north-west portion of Wisconsin and the final two plots located in the southeast and southwest parts of Minnesota and Wisconsin, respectively (Table 1, Fig 4). Most plots in the Lake States region were classified as having low or medium browse impact (Browse Impact Code 2 or 3, Table 1).

**Table 1:** Browse impact assessment for FIA Phase 2-plus plots and number of FIA plots within each state measured between 2012–2015.

Browse Assessment	Browse Impact Score	MI Plots	MN Plots	WI Plots
Very low impact	1	1	0	0
Low impact	2	109	175	92
Medium impact	3	143	90	116
High impact	4	9	21	31
Very high impact	5	0	2	3

Seedling TPH varied more than sapling or overstory TPH for P2 and P2-plus plots. There was a higher maximum in seedling TPH for P2 versus P2-plus plots, but overall standard deviation was less for P2 plots. Variability for seedling TPH was also the highest across the size classes. The mean and standard deviation for overstory BA was nearly the same for both phases, where P2 plots showed a slightly higher maximum (Table 2).

**Table 2:** Summary statistics for forest inventory variables used in randomForest analysis from P2-plus and P2 plots.

Variable	Min	Mean	Max	SD
*P2-plus (n = 786)*				
Seedling TPH	0	7268	55758	7864
Sapling TPH	0	1232	13893	1411
Overstory TPH	0	386	1889	279
Overstory BA (m^2^ha^-1^)	0	16.78	57.96	11.42
*P2 (n = 12*,*386)*				
Seedling TPH	0	5608	116703	6614
Sapling TPH	0	1285	13893	1421
Overstory TPH	0	374	1993	264
Overstory BA (m^2^ha^-1^)	0	16.17	75.63	11.51

**Note:** TPH, trees per hectare; BA, basal area; SD, standard deviation.

Many areas in southwest Minnesota (7/87 counties) and across Michigan (28/83 counties) did not have reported cases of Lyme disease, yet all counties in Wisconsin had at least one reported case from 2012–2015. The greatest number of Lyme disease cases per 10,000 people was Menominee County (22.0 individuals) in Wisconsin, Cass County (20.1 individuals) in Minnesota, and Menominee County (8.6 individuals) in Michigan (Fig 1). Each county across the Lake States had at least one reported deer-vehicle collision from 2012–2015. The greatest number of collisions per 10,000 people occurred in Alcona County (391.8 individuals) in Michigan, Shawano County (180.5 individuals) in Wisconsin, and Lincoln County (44.0 individuals) in Minnesota and minimums of 2.3, 1.8, and 0.6 individuals per 10,000 people in those respective states (Fig 2). From the QDMA deer density map, the maximum possible deer density of >17.4 deer km^-2^ was observed in Wisconsin (59.7% of counties, 89 plots) and Michigan (9.6% of counties, 17 plots). A rare deer density was recorded in two plots located in Wayne County, Michigan (Fig 3).

### Models of deer browse impacts

The most important variables varied for each of the eight models created (Tables 3 and 4). The top three variables for the all states reduced model were deer vehicle collisions, QDMA deer density, and Lyme disease cases with mean variable importances of 34.88%, 26.17%, and 24.31%, respectively. Deer vehicle collisions ranked high in importance for both Michigan and Minnesota models, but the not the Wisconsin models. QDMA deer density estimates ranked high in importance for the Michigan and Wisconsin models, but not the Minnesota models. Sapling TPH had the lowest ranking in importance for all three full models for each of the states (Table 4).

**Table 3:** Models for US Lake States region created using random forests analysis with explanatory variables listed in order of importance predicted by the models.

State	Model	n	Variable	VarImp^2^
All	Full	786		
			Car collisions^1^	16.29
			Deer density	10.39
			Lyme disease cases^1^	8.20
			Overstory BA	7.00
			Sapling TPH	5.54
			Overstory TPH	4.16
			Seedling TPH	2.50
All	Reduced	786		
			Car collisions^1^	34.88
			Deer density	26.17
			Lyme disease cases^1^	24.31

^1^Car collisions and Lyme disease cases are cases per 10,000 people provided by the US census.

^2^VarImp: variable importance–mean decrease in model error from each variable.

**Table 4:** Models for each individual state created using random forests analysis with explanatory variables listed in order of importance predicted by the models.

State	Model	n	Variable	VarImp^2^
MI	Full	260		
			Car collisions^1^	16.19
			Overstory BA	6.05
			Deer density	5.47
			Lyme disease cases^1^	4.15
			Overstory TPH	3.96
			Seedling TPH	3.74
			Sapling TPH	0.81
MI	Reduced	260		
			Car collisions^1^	28.03
			Overstory BA	8.48
			Deer density	6.43
MN	Full	287		
			Lyme disease cases^1^	13.52
			Seedling TPH	9.26
			Car collisions^1^	7.89
			Overstory BA	5.47
			Overstory TPH	2.69
			Deer density	1.27
			Sapling TPH	-1.12
MN	Reduced	287		
			Lyme disease cases^1^	28.61
			Car collisions^1^	23.62
			Seedling TPH	9.70
WI	Full	239		
			Deer density	17.15
			Lyme disease cases^1^	6.69
			Overstory TPH	4.66
			Overstory BA	4.38
			Car Collisions^1^	2.88
			Seedling TPH	2.72
			Sapling TPH	1.54
WI	Reduced	239		
			Deer density	27.61
			Lyme disease cases^1^	14.83
			Car collisions^1^	7.11

^1^Car collisions and Lyme disease cases are cases per 10,000 people provided by the census.

^2^VarImp: variable importance–mean decrease in model error from each variable.

**Fig 5:**
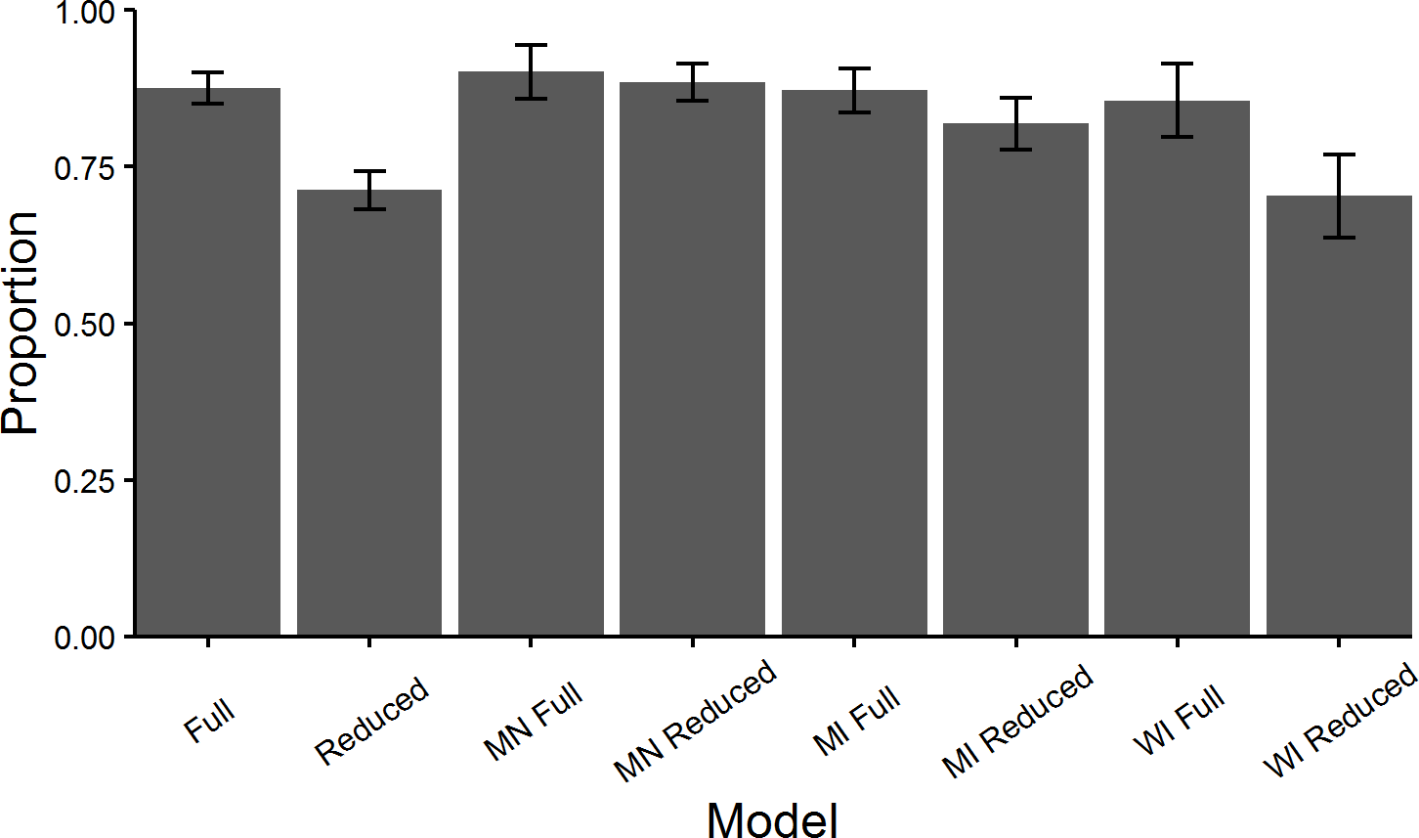
Model accuracy for models created by random forests for the US Lake States. Proportion of plots with browse scores predicted correctly based on models created by random forests models for the Lake States, Minnesota (MN), Wisconsin (WI), and Michigan (MI). Corresponding models found in Tables 3 and 4. Error bars depict ± 1 SD from mean.

The full model for all of the Lake States predicted browse score correctly for 87.62 ± 2.50% (mean ± SD) of plots (Fig 5, Full). The reduced model for combined Lake States predicted browse score correctly for 71.29 ± 3.04% of plots (Fig 5, Reduced). For individual state models, the highest accuracy in the full and reduced models was in Minnesota– 90.18 ± 4.27%, 88.49 ± 2.91% of plots, respectively (Fig 5 –MN Full, MN Reduced). The lowest accuracy for full and reduced models occurred in Wisconsin– 85.58 ± 5.83%, 70.33 ± 6.68% of plots, respectively (Fig 5 –WI Full, WI Reduced).

**Fig 6:**
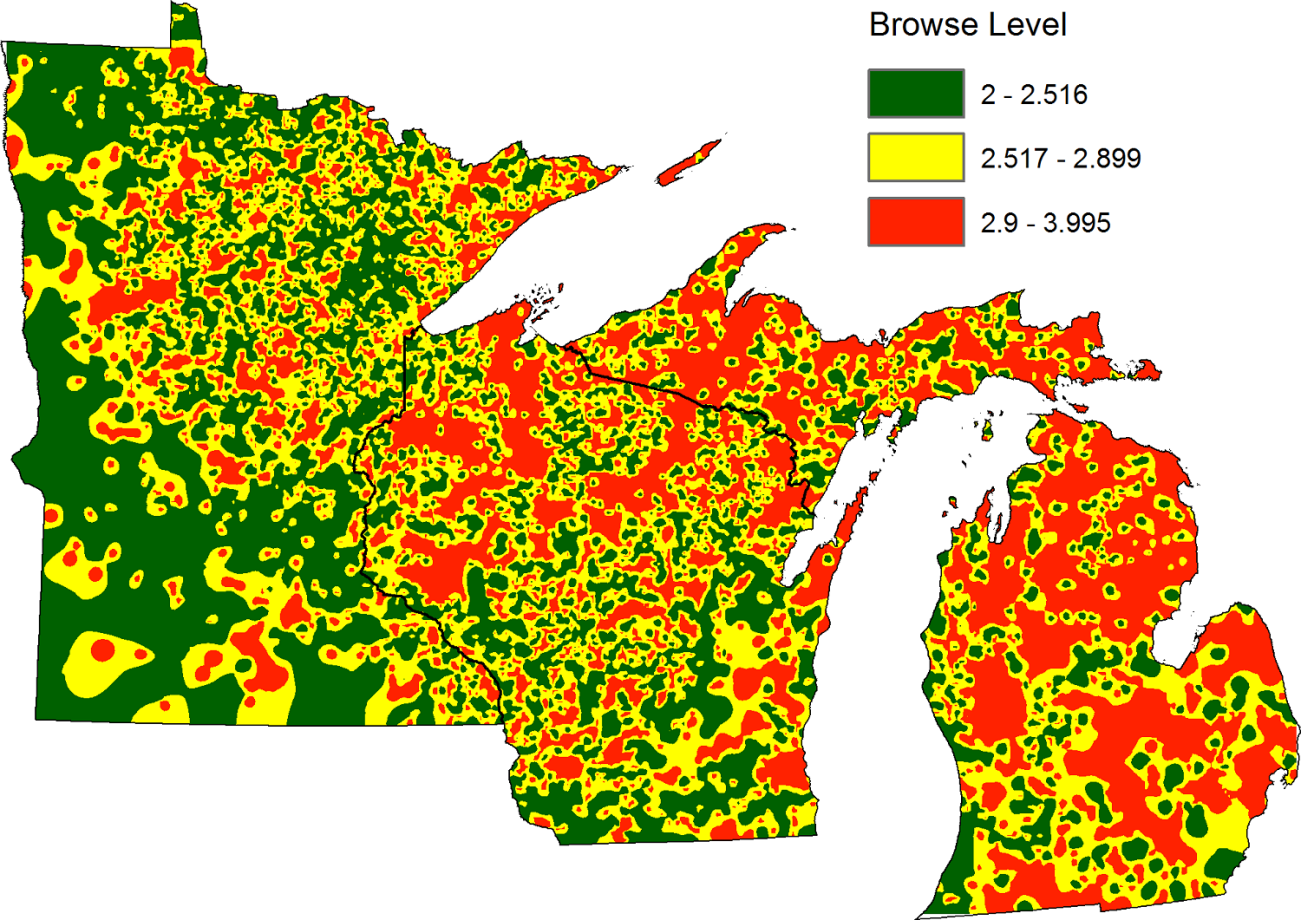
Inverse distance weighted interpolation of deer browse pressure in US Lake States. Browse score based on predicted browse score provided by the full Lake States model. Browse impact score aligns with values described in Table 1. Green indicates the first quantile of predicted browse score, yellow as the second quantile, and red as the third quantile with cutoffs at 0.33 and 0.67 quantiles. White areas indicate insufficient forest cover for prediction.

The interpolated map of Lake States deer browsing indicated most of this region was experiencing some deer browse pressure (second and third quantiles), based on the full model predictions for all states (Fig 6). The second and third quantiles illustrated interpolated browse scores above 2.517, indicating a stronger influence of browse score greater than or equal to 3 in the interpolation, thus corresponding to medium browse impact and higher. The lowest quantile had more influence from a browse score of 2 and was assumed to be low browse impact. In Minnesota, the north-central region as well as a region in the northeast displayed higher browse pressure. The southern half of the state has some areas with browsing, but no major continuous high pressure areas. All of northern Wisconsin displayed a nearly continuous higher browse pressure which is continuous into the Upper Peninsula of Michigan. The Lower Peninsula of Michigan displayed browse pressure across most of the state. This model did not predict the most severe browsing (browse score = 5) for the P2 plot predictions, therefore no plots are represented as higher than 4 on the browse scale (Fig 6).

## Discussion

White-tailed deer populations continue to put stress on forested ecosystems across many areas within the Lake States, hence, understanding their impact on our forests is essential to manage forest resources effectively. This study utilized the FIA database and selected surrogate datasets (Lyme disease occurrence, deer collisions, QDMA deer density estimates) to predict deer browse pressure across the Lakes States with approximately 88% accuracy. The new deer browse indicator recorded by the FIA program provided a nationally consistent method to quantify deer browse pressure across diverse regions and forest types.

The most important variables from the random forests models varied between states. This is likely due to regional differences in forest types and management strategies across the Lake States. Additionally, deer density estimates provided by QDMA were derived from state-specific criteria and the results may reflect these differences [40]. Deer-vehicle collisions were consistently one of the top predictors for all eight models created. All counties had at least one reported deer-vehicle collision from 2012–2015, hence this consistent coverage likely captured the variation across the region well. While deer collisions may be under-reported across all states (e.g. Minnesota had 2,096 reported deer-vehicle collisions in 2014, but State Farm Insurance projected 37,500 total deer-vehicle collisions in that year (2015)), the proportion of collisions that are reported may be expected to be consistent within each state.

Lyme disease cases also displayed high importance in most of the random forests models. The full Michigan model placed Lyme disease cases lower than that of the other models, but many counties across Michigan reported little to no cases of Lyme disease from 2012–2015. Without consistent coverage, this may have reduced the variable importance for Lyme disease in Michigan. In contrast, Minnesota and Wisconsin have more reported cases of Lyme disease than Michigan with coverage across most or all of the counties. Possible under-reporting or misclassification of Lyme disease as well as the classification of county of residence, not county of exposure potentially limit the Lyme disease dataset [46]. The vector for this disease, the black-legged tick, has a variety of vertebrate hosts other than white-tailed deer, primarily *P*. *leucopus* (white-footed mouse), which is distributed across the lower peninsula of Michigan and in much of Wisconsin and Minnesota [47]. Populations of *P*. *leucopus* vary greatly temporally and regionally [48], which may contribute to some variability in tick distribution. Most research on *P*.*leucopus* populations contributing to Lyme disease have been in the eastern US with limited studies in the Lake States; however, we assumed that it functions equally as a black-legged tick host in the Lake States [47,49–51]. Our models support the importance of using Lyme disease information when estimating deer browsing across regional scales, especially in areas with consistent reporting of Lyme disease cases.

Deer browse pressure is predicted to occur across most of the Lake States region with forested cover, as seen in the yellow and red areas in Fig 6. Given the spatial scale and number of plots available for analysis, this map may be interpreted as a coarse estimate of deer browse impact and does not exactly represent the discrete categories described in McWilliams et al. [35]. Given the influence of nearby plots on interpolated space, the lowest quantile is assumed to be similar to low deer impact (Table 1) and the other two quantiles describe increasing levels of browse above low impact. Southern Minnesota is dominated by prairie or agricultural land and does not have many FIA plots contributing to the interpolation. The heterogeneity of agricultural and forested land in southern Minnesota has the potential to support a high deer population not captured by this dataset. The primary predator of white-tailed deer today is humans, but wolves (*Canis lupus*), coyotes (*Canis latrans*), bobcats (*Lynx rufus*), cougars (*Puma concolor*) and black bears (*Ursus americanus*) can also prey on white-tailed deer. Wolves, bobcats, cougars and bears have migrated away from urban and suburban landscapes, but in northeastern Minnesota, northern Wisconsin, and the Upper Peninsula of Michigan, there is a higher predator population potentially contributing to the control of white-tailed deer populations and regulating the browse pressure in those areas [30]. In some predator occupied areas, predicted deer browse pressure exists in the highest quantile due to the heterogeneity of predators across the landscape at this spatial scale (Fig 6). This patchiness has been observed in northern Wisconsin with wolf predation affecting deer populations and vegetation response in browse-sensitive white cedar wetlands, although presence or absence of predators only partially addresses a vegetation response at lower tropic levels [52,53].

The societal costs of high deer populations from DVC and vectoring Lyme disease is compounded with the financial burden of managing deer populations and their impacts at the forest level [54]. Properly constructed deer exclosures are effective in excluding deer from an area (e.g. [13,55–57]), but they can have substantial financial costs in implementation and maintenance and have limited applicability across large spatial scales. Bud capping is also relatively successful in protecting conifer seedlings and saplings from deer browsing, but requires numerous labor hours across multiple years to be effective. Hunting is an important deer management tool in the Lake States, particularly in areas with high human populations. Forested communities have exhibited recovery of preferred browse species following managed deer hunting, which supports hunting as an effective management tool [58,59]. Understanding deer browse pressure can allow for forest management that can be economically, socially, and ecologically successful.

The browse metric provided by the FIA program is superior to deer density in the forestry context, because tree seedling abundance in relation to deer densities alone varies in the eastern and Midwestern US. While palatable tree species regeneration generally declines with deer densities between 4 and 5.8 deer per square kilometer and above [60,61], some forest types such as those dominated by oak display both high deer density concomitant with high seedling abundance, a reflection of past forest management and competing vegetation. This deer browse metric provides an opportunity to explore potential effects on the forested community at different levels of deer browse pressure, which may or may not be proportional to deer density. The map of estimated browse impacts created from this modeling effort (Fig 6) is designed to provide a tool for land managers to utilize–in conjunction with other criteria such as topography, palatable species abundance, and deer winter cover–as a means of coarsely estimating deer browse pressure within forested landscapes in the Lake States. Browse susceptible areas on the map indicate areas of emphasis where further investigation into deer browse severity may be a financially viable option. In contrast, low browse impact areas may not need direct management to regenerate and establish trees that are preferred species for deer (e.g., northern white cedar (*Thuja occidentalis*), yellow birch (*Betula alleghaniensis*), eastern white pine (*Pinus strobus*), eastern hemlock (*Tsuga canadensis*), and northern red oak (*Quercus rubra*) in the Lake States [5]). Future work may address other related community effects of deer browse such as invasive species abundance and herbaceous vegetation response with this new deer browse metric. This study displays the important link between ecological data and societal information in natural resource management, which is beneficial for outreach and education of a polarizing topic. With future data from P2-plus plot measurements from the FIA program, this model can be refined to better quantify browse impacts on forests across the Lake States.

## Supporting information

S1 Table Summary of browse score by county for the Lake States (US). (PDF)
