# Supplementary material for: Quantifying impacts of white-tailed deer (Odocoileus virginianus Zimmerman) browse using forest inventory and socio-environmental datasets
Source: PLoS One. 2018 Aug 23;13(8):e0201334. doi: 10.1371/journal.pone.0201334 (PMC6107117; doi:10.1371/journal.pone.0201334)
Supplement: S1 Table — (PDF) [file pone.0201334.s001.pdf]

S1 Table

State	County	min	max	n
Michigan	Alcona	3	3	3
Michigan	Alger	2	3	6
Michigan	Allegan	2	2	6
Michigan	Alpena	2	2	2
Michigan	Antrim	3	3	2
Michigan	Arenac	3	3	1
Michigan	Baraga	2	3	5
Michigan	Barry	2	2	1
Michigan	Berrien	2	2	1
Michigan	Branch	2	2	1
Michigan	Calhoun	2	2	2
Michigan	Charlevoix	3	3	1
Michigan	Cheboygan	2	3	6
Michigan	Chippewa	2	3	11
Michigan	Clare	3	3	4
Michigan	Clinton	3	3	1
Michigan	Crawford	2	3	5
Michigan	Delta	2	4	13
Michigan	Dickinson	2	3	5
Michigan	Eaton	2	2	2
Michigan	Emmet	3	3	3
Michigan	Genesee	3	3	1
Michigan	Gladwin	3	4	5
Michigan	Gogebic	3	3	5
Michigan	Grand Traverse	3	3	3
Michigan	Houghton	2	4	8
Michigan	Huron	3	3	1
Michigan	Ingham	2	2	2
Michigan	Iosco	3	3	4
Michigan	Iron	2	3	9
Michigan	Isabella	2	2	1
Michigan	Kalamazoo	2	2	2
Michigan	Kalkaska	2	3	7
Michigan	Kent	3	3	1
Michigan	Keweenaw	3	3	4
Michigan	Lake	3	3	4
Michigan	Leelanau	2	3	4

Michigan	Livingston	2	2	2
Michigan	Luce	2	4	12
Michigan	Mackinac	2	4	11
Michigan	Manistee	3	3	3
Michigan	Marquette	2	3	14
Michigan	Mason	2	2	1
Michigan	Mecosta	3	3	3
Michigan	Menominee	2	3	6
Michigan	Midland	3	3	1
Michigan	Missaukee	3	3	2
Michigan	Montcalm	3	3	1
Michigan	Muskegon	2	3	2
Michigan	Newaygo	2	3	5
Michigan	Ogemaw	2	3	3
Michigan	Ontonagon	2	3	10
Michigan	Osceola	2	3	5
Michigan	Oscoda	3	3	1
Michigan	Otsego	2	3	5
Michigan	Ottawa	2	4	4
Michigan	Presque Isle	2	3	3
Michigan	Roscommon	2	3	4
Michigan	Saginaw	1	3	3
Michigan	Schoolcraft	2	3	5
Michigan	Shiawassee	2	2	1
Michigan	St. Clair	2	2	1
Michigan	Tuscola	3	3	1
Michigan	Van Buren	2	2	3
Michigan	Washtenaw	2	2	2
Michigan	Wayne	2	2	2
Michigan	Wexford	3	3	4
Minnesota	Aitkin	2	4	12
Minnesota	Anoka	2	4	2
Minnesota	Becker	2	3	8
Minnesota	Beltrami	2	5	14
Minnesota	Benton	3	3	1
Minnesota	Brown	2	2	1
Minnesota	Carlton	3	4	5
Minnesota	Cass	2	4	15
Minnesota	Chisago	4	4	1

Minnesota	Clearwater	2	2	6
Minnesota	Cook	2	3	12
Minnesota	Crow Wing	2	4	7
Minnesota	Dakota	2	2	1
Minnesota	Dodge	2	2	1
Minnesota	Goodhue	2	3	3
Minnesota	Grant	2	2	1
Minnesota	Hennepin	2	2	1
Minnesota	Houston	3	3	3
Minnesota	Hubbard	2	4	12
Minnesota	Itasca	2	4	21
Minnesota	Kanabec	2	3	3
Minnesota	Kandiyohi	2	2	1
Minnesota	Kittson	3	3	1
Minnesota	Koochiching	2	3	23
Minnesota	Lac qui Parle	2	2	1
Minnesota	Lake	2	3	20
Minnesota	Lake of the Woods	2	4	8
Minnesota	Le Sueur	2	2	1
Minnesota	Mahnomen	4	4	1
Minnesota	Martin	2	2	1
Minnesota	McLeod	3	3	1
Minnesota	Mille Lacs	2	2	3
Minnesota	Morrison	2	4	3
Minnesota	Olmsted	3	3	1
Minnesota	Otter Tail	2	4	5
Minnesota	Pine	2	3	8
Minnesota	Polk	2	2	3
Minnesota	Rice	2	2	1
Minnesota	Roseau	2	3	4
Minnesota	Sherburne	2	3	3
Minnesota	St. Louis	2	4	48
Minnesota	Stearns	3	4	2
Minnesota	Stevens	2	2	1
Minnesota	Swift	2	4	2
Minnesota	Todd	2	4	4
Minnesota	Wabasha	2	3	2
Minnesota	Waseca	2	2	1
Minnesota	Washington	3	3	1

Minnesota	Winona	2	5	5
Minnesota	Wright	2	3	2
Minnesota	Yellow Medicine	2	2	1
Wisconsin	Adams	2	3	5
Wisconsin	Ashland	2	5	10
Wisconsin	Barron	2	3	2
Wisconsin	Bayfield	2	5	9
Wisconsin	Brown	2	2	2
Wisconsin	Buffalo	2	2	2
Wisconsin	Burnett	2	4	9
Wisconsin	Chippewa	2	4	5
Wisconsin	Clark	2	3	4
Wisconsin	Columbia	2	2	1
Wisconsin	Crawford	3	5	2
Wisconsin	Dane	2	2	1
Wisconsin	Dodge	2	2	2
Wisconsin	Door	2	2	2
Wisconsin	Douglas	2	4	9
Wisconsin	Dunn	2	3	4
Wisconsin	Eau Claire	2	3	6
Wisconsin	Florence	3	3	3
Wisconsin	Fond du Lac	2	2	2
Wisconsin	Forest	3	4	5
Wisconsin	Grant	2	3	2
Wisconsin	Iowa	3	3	3
Wisconsin	Iron	2	3	6
Wisconsin	Jackson	2	2	6
Wisconsin	Juneau	2	2	3
Wisconsin	Kenosha	2	3	2
Wisconsin	La Crosse	2	3	3
Wisconsin	Langlade	3	3	6
Wisconsin	Lincoln	2	4	8
Wisconsin	Manitowoc	2	2	1
Wisconsin	Marathon	2	3	6
Wisconsin	Marinette	2	4	14
Wisconsin	Marquette	2	4	2
Wisconsin	Menominee	2	2	2
Wisconsin	Monroe	2	3	2
Wisconsin	Oconto	3	3	6

Wisconsin	Oneida	2	4	7
Wisconsin	Outagamie	2	2	1
Wisconsin	Pepin	4	4	2
Wisconsin	Pierce	2	2	1
Wisconsin	Polk	3	3	2
Wisconsin	Portage	2	2	4
Wisconsin	Price	2	4	7
Wisconsin	Richland	2	3	3
Wisconsin	Rock	3	3	1
Wisconsin	Rusk	3	4	7
Wisconsin	Sauk	2	3	4
Wisconsin	Sawyer	2	4	11
Wisconsin	Shawano	2	4	2
Wisconsin	Sheboygan	2	2	1
Wisconsin	St. Croix	2	2	1
Wisconsin	Taylor	3	4	6
Wisconsin	Trempealeau	2	3	5
Wisconsin	Vernon	2	4	2
Wisconsin	Vilas	3	3	4
Wisconsin	Walworth	2	2	1
Wisconsin	Washburn	2	4	4
Wisconsin	Washington	3	3	1
Wisconsin	Waukesha	3	3	1
Wisconsin	Waupaca	2	3	2
Wisconsin	Waushara	3	3	1
Wisconsin	Wood	2	2	4
